# Efficacy of NH_3_ as a secondary barrier treatment for inactivation of *Salmonella* Typhimurium and methicillin-resistant *Staphylococcus aureus* in digestate of animal carcasses: Proof-of-concept

**DOI:** 10.1371/journal.pone.0176825

**Published:** 2017-05-05

**Authors:** Jacek A. Koziel, Timothy S. Frana, Heekwon Ahn, Thomas D. Glanville, Lam T. Nguyen, J. (Hans) van Leeuwen

**Affiliations:** 1 Dept. of Agricultural and Biosystems Engineering, Iowa State University, Ames, Iowa, United States of America; 2 Dept. of Civil, Construction and Environmental Engineering, Iowa State University, Ames, Iowa, United States of America; 3 Dept. of Food Science and Human Nutrition, Iowa State University, Iowa State University, Ames, Iowa, United States of America; 4 Dept. of Veterinary Diagnostic and Production Animal Medicine, Iowa State University, Ames, Iowa, United States of America; 5 Dept. of Animal Biosystems Science, Chungnam National University, Daejeon, Republic of Korea; Animal and Plant Health Agency, UNITED KINGDOM

## Abstract

Managing the disposal of infectious animal carcasses from routine and catastrophic disease outbreaks is a global concern. Recent research suggests that burial in lined and aerated trenches provides the rapid pathogen containment provided by burial, while reducing air and water pollution potential and the length of time that land is taken out of agricultural production. Survival of pathogens in the digestate remains a concern, however. A potential answer is a ‘dual’-barrier approach in which ammonia is used as a secondary barrier treatment to reduce the risk of pathogen contamination when trench liners ultimately leak. Results of this study showed that the minimum inhibitory concentration (MIC) of NH_3_ is 0.1 M (~1,468 NH_3_-N mg/L), and 0.5 M NH_3_ (~7,340 NH_3_-N mg/L) for ST4232 & MRSA43300, respectively at 24 h and pH = 9±0.1 and inactivation was increased by increasing NH_3_ concentration and/or treatment time. Results for digestate treated with NH_3_ were consistent with the MICs, and both pathogens were completely inactivated within 24 h.

## Introduction

### Outbreaks of infectious animal diseases

It is estimated that in the U.S. 1.4 billion metric tons of routine livestock mortalities and 0.16 billion metric tons of routine poultry losses were disposed in 2000 [1]. Emergency disposal rates can be several orders of magnitude greater during natural disasters or intentional or accidental introduction of infectious animal disease [2]. Bacterial pathogens contribute to animal disease outbreaks [3–5] and human health concerns [6–8]. There is a growing body of evidence that livestock animals can be reservoirs of infectious foodborne diseases that pose risks to susceptible human populations [9–16]. Therefore, effective disposal of potentially infectious animal mortalities is a key component of a successful response to a disease outbreak and routine livestock production management [17–19].

### Carcass disposal methods

Carcass disposal methods include burial, composting, incineration, commercial landfills, rendering, and alkaline hydrolysis [20]. Burial, composting, incineration, and rendering are commonly used for disposal of relatively small numbers of mortalities that occur routinely during production. However, in emergency situations, such as disease outbreaks, fire, flooding, and hurricanes, disposal is greatly complicated by the need to deal with large numbers of mortalities within a short time-frame to address biosecurity, transportation logistics, public perception, and environmental concerns. In such situations, the preferred methods for disposal of animal mortalities are an on-farm burial and on-farm composting. Both minimize biosecurity and environmental risks by rapidly sequestering infected and decaying mortalities at their source instead of transporting them to off-site disposal facilities [2,20]. Of the two, burial is more common than composting as it is much faster and does not require sourcing of large quantities of cover material or disposal of post-treatment residues [1, 21]. However, leachate from burial sites can cause chemical and microbial contamination of groundwater due to poor site selection or improper construction [22]. In some instances, emergency burial has also resulted in complaints about odor. Long-term loss of agricultural land use [23] is also a concern since carcass decay in burial sites is slow and undigested whole carcasses can be found at burial sites years after emergency disposal [21].

### Burial-aerobic digestion hybrid concept for in-trench, on-farm carcass disposal

Following a widespread outbreak of Foot-and-Mouth disease in 2010, the Korean government (Rural Development Administration, National Institute of Animal Science) sponsored studies of several enhanced carcass disposal methods designed to overcome serious public concerns regarding odor, groundwater pollution potential; and loss of productive land due to slow carcass decomposition within burial plots. One of the proposed methods combines on-farm burial in trenches lined with impermeable fabric, with in-trench aeration to accelerate decomposition and reduce the contamination potential of liquid digestion products.

Phase 1 of the evaluation of this strategy [24] involved lab-scale studies evaluating: the ability of aerobic digestion (AeD) to accelerate carcass decay; and of the potential to use volatile organic compounds (VOCs) released during aerobic digestion as a biosecure way to assess the degree of carcass decomposition without removing digestate or solids from the trench. Results of these preliminary studies showed a reduction of biochemical oxygen demand (BOD, 99.9%), volatile suspended solids (VSS, 99.2%), and total suspended solids (TSS, 99.1%) resulting in digestate meeting the U.S. Environmental Protection Agency (EPA) wastewater disposal criteria. Also, a significant reduction (>6-log) of model *Salmonella* and *Staphylococcus* after week 1 and week 4, respectively were observed [24], thus, exemplifying the efficacy of the primary barrier treatment. Furthermore, carcass decomposition using AeD was nearly complete (95%) in about 13 weeks while decomposition of similarly sized carcasses using anaerobic digestion was negligible. However, concerns remain regarding pathogen release should the trench liner leak or if it becomes necessary to pump digestate from the trench and dispose of it elsewhere. Thus, the motivation to test a secondary barrier treatment, in conjunction with the burial-AeD hybrid concept, to further reduce the risk of pathogen re-emergence.

Evidence of AeD performance for treatment of animal and poultry carcasses is still fairly limited. Aerobic digestion was first studied in the UK as a novel technology option for temporary storing and pretreating of sheep carcasses prior to final disposal [25, 26]. It was reported that bacterial counts (*Salmonella enterica* (*S*. *enterica*, serotype Senftenberg and Poona), *Enterococcus faecalis* (*E*. *faecalis*), *Campylobacter jejuni* (*C*. *jejuni*), *Campylobacter coli* (*C*. *coli*), and *Escherichia coli* (*E*. *coli*) O157) in sheep carcass components, including muscle, bone, fat, pelt, blood, stomach contents, wool, and liver, decreased significantly (>5-log values from the original starting concentration) during ~3 month retention in an AeD process [26]. However, *E*. *faecalis* remained detectable until the end of 3 months of the trial [26]. These bacteria reported by Gwyther et al. [26] are also important foodborne bacteria monitored by the U.S. Food & Drug Administration [27].

### Pathogen survival potential suggests the need for a secondary disinfection strategy for carcass disposal

*Salmonella* spp. and *Staphylococcus* spp. are common bacteria found in animals which may also be pathogens and potential zoonotic agents with the capacity to adapt and survive in a wide variety of different foods and environments [28, 29]. They are representative of a broad category of foodborne pathogens related to infections of humans and animals [30, 31] and are often present in poultry both externally (surface of the body) and intestinally (gastrointestinal system) [32]. Some researchers have suggested that pathogenic bacteria and other microorganisms in manure residues pose potential risks to human and animal health, and the environment [33–35]. Similarly, the biosecurity risk associated with the use of untreated digestate residues as fertilizer for farmland is difficult to assess, but this risk cannot be neglected [36]. Therefore, there is a need to investigate a secondary barrier approach (e.g.) disinfection of digestate residues with appropriate chemicals for burial-AeD treatment of animal mortalities. *Salmonella* spp. and *Staphylococcus* spp. represent different bacterial groups (gram-positive and gram negative). Therefore, the effect of disinfection can be somewhat assessed across gram-positive and gram-negative organisms.

### Evidence of ammonia as an inactivating agent

While there are many possible (and stronger) disinfectants, ammonia has advantages for the on-farm treatment application. Ammonia is readily available at the relatively low cost in agricultural regions where it is a commonly used crop fertilizer. Widespread experience with handling and using ammonia in farming communities is also an important advantage in emergency situations. Ammonia is one of the products of aerobic and anaerobic digestion of organic nitrogen [37]. These two biological processes are the most common methods used to treat animal and human waste [38–41]. Ammonia is generated through ammonification during decomposition of organic matter rich in nitrogen [42]. Depending on the concentration, pH, and temperature, ammonium (NH_4_^+^) serves as a beneficial nutrient or ammonia (NH_3_) serves as a toxicant to various waterborne organisms [43–46]. Ammonia, the bactericidal form, so-called ‘un-ionized NH_3_^’^, or ‘free NH_3_’, increases in water solution with increasing pH level and starts to dominate at a pH > pKa or ~9 [47].

Ammonia emitted from animal waste has been reported to inactivate several common bacterial pathogens [48] including *Salmonella* Typhimurium (ST), *E*. *coli* O157:H7, and *Listeria monocytogenes* (*L*. *monocytogenes*) in manure [49]. It also has been proposed that ammonization could be used as a disinfection process of community sewage sludge [50]. Fumigation with ammonia has been applied to inactivate *E*. *coli* O157:H7 and ST in alfalfa seeds and mung beans [51]. Injections of gaseous NH_3_ has been used to kill pathogens, included *E*. coli O157:H7, *L*. *monocytogenes*, and *S*. *enterica*, on boneless lean beef trimmings [52]. Ammonia also has been used to disinfect zoonotic bacteria such as *S*. Newport, *C*. *jejuni*, *E*. *coli* O157:H7, *L*. *monocytogenes*, and *Yersinia enterocolitica* (*Y*. *enterocolitica*) in animal feed [53]. To date, however, there is no research on ammonia disinfection of digestate residues remaining after AeD of infected animal carcasses containing pathogenic bacteria.

### Study objectives

The objectives of this study were: (1) to determine the minimum inhibitory concentrations (MICs) of NH_3_ for *Salmonella* Typhimurium and *Staphylococcus aureus* in a laboratory setting and (2) to evaluate the efficacy of the previously determined NH_3_ MIC to inactivate these pathogens in a chemically- and microbially- complex digestate matrix of aerobically digested poultry carcasses inoculated with these two pathogens. Marker strains were used to improve their detection and quantification in a microbially complex digestate matrix. Our working hypothesis is that the secondary barrier treatment with NH_3_ will significantly reduce the level of infectious bacteria (1) during aerobic digestion (early-phase AeD) and (2) after aerobic digestion is complete (late-phase AeD). A success of secondary barrier treatment in early-phase AeD can provide useful information if time and resources can be potentially saved by applying NH_3_ treatment earlier. Treatment with NH_3_ in the early-phase AeD is a critical challenge to the NH_3_ treatment concept. If it worked under those complex conditions, it was likely to work under less difficult conditions. Post-digestion treatment of digestate with NH_3_ could conceivably become a useful addition to the burial-AeD disposal method, thereby making it more biosecure.

## Materials and methods

The rationale was to test the secondary barrier approach concept in which NH_3_ is used as a secondary barrier treatment for further development of feasible solutions for biosecure emergency disposal of infectious carcasses. The MIC (Objective 1) was a laboratory test that was designed to reflect practical field conditions. Results from Objective 1 were then applied (time and dose) to a chemically- and microbially-complex digestate matrix (Objective 2).

### Overall experimental design including primary and secondary barrier treatments

**Fig 1:**
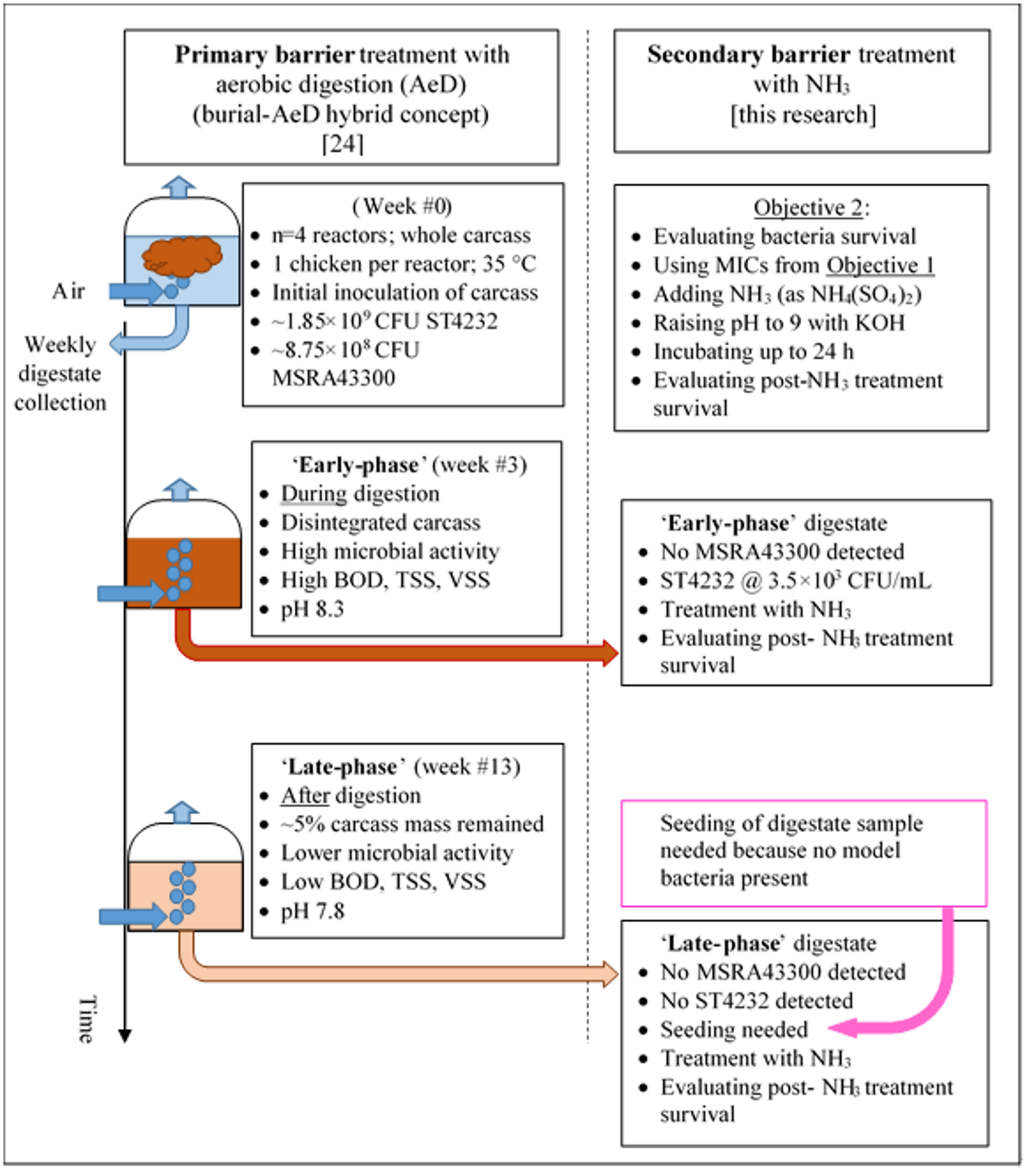
Overall experimental design including primary and secondary barrier treatments.

Poultry was used as a model carcass source. White Leghorn (*Gallus gallus domesticus*) were raised at the ISU Poultry Research and Teaching facility and were euthanizing under IACUC log #4-03-5425-G. Weekly collection of digestate samples from each (n = 4) reactor was subjected to incubation and bacterial enumeration, measuring pH, BOD, TSS, VSS, dissolved oxygen with standard methods described in detail elsewhere [24]. Fig 1 summarizes key methodology details differentiating the primary and secondary barrier treatment research.

### Model bacterial strains

In this current study, two available marker strains were used with selective growth media that suppresses non-study microorganisms in the liquid digestate samples. For this practical reason, nalidixic acid-resistant ST4232 was obtained from the U.S. Department of Agriculture—Agricultural Research Service (USDA-ARS, Ames, Iowa), and MRSA43300 was obtained from the Dept. of Veterinary Diagnostic and Production Animal Medicine (VDPAM), Iowa State University (ISU). The handling of bacterial strains was approved by the IBC 11-I-0030-A/H project ‘Development of environmentally friendly livestock mortality disposal systems using aerobic digestion.'

To prepare for inoculation (of either the phosphate buffered saline (PBS) or digestate), each selected bacterial species was cultivated separately overnight on different blood agar plates (TSA with 5% sheep blood) (Remel, Lenexa, KS). *Salmonella* and *Staphylococcus* suspensions were prepared by thoroughly mixing 3 to 5 colonies of each selected bacteria in separated 15 mL sterile screw-capped glass tubes containing 5 mL of PBS solution (Becton Dickinson, Franklin Lakes, NJ). McFarland turbidity standard No. 0.5 (Becton Dickinson, Franklin Lakes, NJ) was used as a reference to adjust the turbidity of bacterial suspensions. The concentration of each bacterial suspension was estimated to be approximately 1 × 10^8^ CFU/mL with final concentrations determined using standard plate counts.

Chrome agar (Bio-Rad Laboratories Inc., Hercules, CA) and XLT4-nalidixic acid agar (prepared and provided by VDPAM, ISU) were used for ST4232 and MRSA43300 enumeration. The number of model bacteria in each sample was obtained by spreading 100 μL of 10-fold serial dilutions (10^0^ to 10^−8^) on selective media, and then incubating at 35°C for 48 h. Total colony counts were enumerated using a Q-Count Automated Colony Counter System (Spiral Biotech Advanced Instruments Inc., Norwood, MA). Bacterial colonies from XLT4-nalidixic acid agar or chrome agar were selected and identification confirmed using matrix—assisted laser desorption-ionization time-of-flight mass spectrometry (MALDI-TOF-MS) Biotyper System (Bruker Corp., Fremont, CA).

### Determination of the minimum inhibitory concentrations of NH_3_

*Objective* #1: Determination of the MICs of ammonia for inactivation of ST4232 and MRSA43300. Several lessons were learned while conducting preliminary treatment tests at mid-to-low pH (4.4 to 4.6) in sterile saline solution to observe the effects of NH_3_ dose (NH_3_ source was NH_4_Cl) and time (24, 48, and 72 h) on *Salmonella*. Due to low pH, there was no NH_3_, i.e., an NH_3_-N fraction of the total ammoniacal nitrogen (TAN) ranging from 0 to 0.001 at 12.5 to 15.9°C. (NH_3_-N is just an expression for measurement of NH_3_ in water solution.) More importantly, there was no effect on *Salmonella* growth at low pH (S1 Fig).

Due to the lack of effect at low initial pH = 4.4 to 4.6 and relatively low temperatures, new experiments were conducted at a pH 9.0±0.1, close to the pKa of TAN, to about half of the NH_3_-N fraction of TAN at 35°C [54]. Temperature itself can boost the NH_3_-N fraction of TAN [54]. Three replicates of treatment and control were used. (NH_4_)_2_SO_4_ was added to achieve a desired, nominal [NH_3_] of 0.05 M, 0.1 M, 0.3 M, and 0.5 M. More accurate concentrations and the bactericidal NH_3_ molar fractions are reported in Table 1. TAN and [NH_3_-N] were confirmed using Standard Methods [55] #4500-NH_3_-N. No (NH_4_)_2_SO_4_ was added into the controls. While there is a consensus that NH_3_ is bactericidal, there is not much known about the potentially toxic role of sulfate. To the contrary, sulfate-reducing bacteria are thought to be beneficial to environmental cleanup for metals and hydrocarbons. The pH of the experimental liquid was adjusted with potassium hydroxide (KOH) to 9.0 ± 0.1. The caps of tubes were screwed tight to protect the experimental liquid and to prevent loss of NH_3_ gas to the atmosphere. All experimental tubes were incubated at 35°C during four different treatment times (0.5 h, 4 h, 8h, and 24 h). Surviving numbers of the two model bacteria were determined using the enumeration procedure described above.

**Table 1:** Summary of ammonia treatment concentrations.

Nominal* **[NH**_**3**_**]**	**0.05 M**	**0.1 M**	**0.3 M**	**0.5 M**
Actual** **[NH**_**3**_**] (M)**	0.0524	0.105	0.313	0.524
**pH (+/- 0.1)**	9	9	9	9
**T (°C)**	35	35	35	35
**p*Ka***	8.949	8.949	8.949	8.949
**Molar fraction of NH**_**3**_**-N (-)**	0.529	0.529	0.529	0.529
**NH**_**3**_**-N (mg/L)**	734	1,468	4,404	7,340
**NH**_**4**_**-N (mg/L)**	653	1,307	3,921	6,535
**TAN [NH**_**3**_**-N + NH**_**4**_**-N] (mg/L)**	1,387	2,775	8,325	13,875
**TAN (M)**	0.0991	0.1982	0.5946	0.9911

*Nominal concentrations in this manuscript are based on rounded-off single significant figure [NH_3_].

**Actual [NH_3_] (M) rounded-off to 3 significant figures.

The following formula was used to calculate ammonia concentrations: *f* = 1/[10^(p*Ka*-pH) + 1]; where: p*Ka* = 0.0901821 + (2729.92/*T*); *f* = mole fraction of NH_3_-N; *T* = temperature (K) [54].

### NH_3_ treatment after the 1^st^ barrier-treated digestate

The resulting MIC informed experimental design for Objective #2, i.e., ‘secondary barrier’ treatment of digestate at two stages of the digestion process, i.e. the ‘early-’ and ‘late-phase’ case scenarios, respectively. The ‘early-phase’ scenario represented a high microbial activity during aerobic digestion (week #3, high BOD, highest TSS & VSS levels, and the first visual evidence of the whole carcass breakup and disintegration) and the ‘late-phase’ scenario when aerobic digestion is complete (week #13, represented by lower microbial activity post the 99% reduction of BOD, TSS, and VSS, respectively) [24].

#### Testing secondary barrier treatment in the ‘early-phase’ AeD scenario

The ‘early-phase’ AeD scenario represented an opportunity to test the secondary barrier approach at a period of high microbial activity (initial first few weeks) while the carcass digestion was still in progress. Visual observations of process confirmed that the carcasses disintegrated spilling internal organs into the digestate [24] at about week #3. In this trial, a 5-mL aliquot of the 3^rd^ week digestate was withdrawn directly from each (n = 4) aerobic reactor, dispensed into 15 mL tubes and cultured for surviving ST4232 and MRSA43300. These model bacteria were initially inoculated into chicken carcasses at week #0 (1.85 × 10^9^ CFU/carcass (ST4232) and 8.75 × 10^8^ CFU/carcass (MSRA)) and monitored weekly for concurrent (proof-of-concept, primary barrier treatment only) study [24]. At week #3, MRSA was not recovered; however, ST4232 was recovered and enumerated (3.5 × 10^3^ CFU/mL). Concurrently, an amount of (NH_4_)_2_SO_4_ was added into each treatment tube to obtain NH_3_ = 0.1 M (i.e. the MIC concentration consistent with the results from testing Objective #1 for ST4232). No (NH_4_)_2_SO_4_ was added to the control tubes. Then, the pH of digestate samples was adjusted with KOH to 9.0±0.1, capped tight, and incubated at 35°C during four different treatment times (0.5 h, 4 h, 8h, and 24 h). Four replicates with digestate from separate reactors were treated with NH_3_. The bacterial counts were determined using a similar procedure as described earlier.

#### Testing secondary barrier treatment in the ‘late-phase’ AeD scenario

The ‘late-phase’ AeD scenario represented an opportunity to test the secondary barrier approach at a period of lower microbial activity at the end of digestion (week #13) when only digested feather and bone fragments were left in the digestate [24]. At week #13, neither ST4232 nor MRSA43300 were recovered. Thus, both model bacteria were seeded into digestate samples before NH_3_ treatment. To make 100 mL of the mixture with 1 × 10^6^ CFU/mL ST4232 and MRSA43300 concentrations, an amount of 99 mL of digestate sample was withdrawn from each reactor and mixed with 1 mL of each pure selected bacterial suspension (approximately 1 × 10^8^ CFU/mL). Inoculated digestate samples were treated with (NH_4_)_2_SO_4_ at two different NH_3_ concentrations (0.1 M, and 0.5 M, i.e. MIC concentrations consistent with results for Objective #1) with 4 treatment times (0.5 h, 4 h, 8 h, and 24 h). No (NH_4_)_2_SO_4_ was added into the controls. The pH adjustment to 9.0±0.1, tube capping, incubation temperature, and enumeration procedure was identical as described earlier.

### Statistical analyses

Pathogen concentrations were analyzed for the effects of ammonia using the one-way analysis of variance (ANOVA) method. The significance of differences in pathogen levels between control and treated samples was tested using the Tukey-Kramer HSD (honestly significant difference) test at *p* ≤ 0.05. Microbiological data were transformed into log_10_ to avoid analyzing data with zero values. All computations were carried out using SigmaPlot v11.0.0.77 (Systat Software Inc., San Jose, CA).

## Results and discussion

### Minimum inhibitory concentration of NH_3_

**Fig 2:**
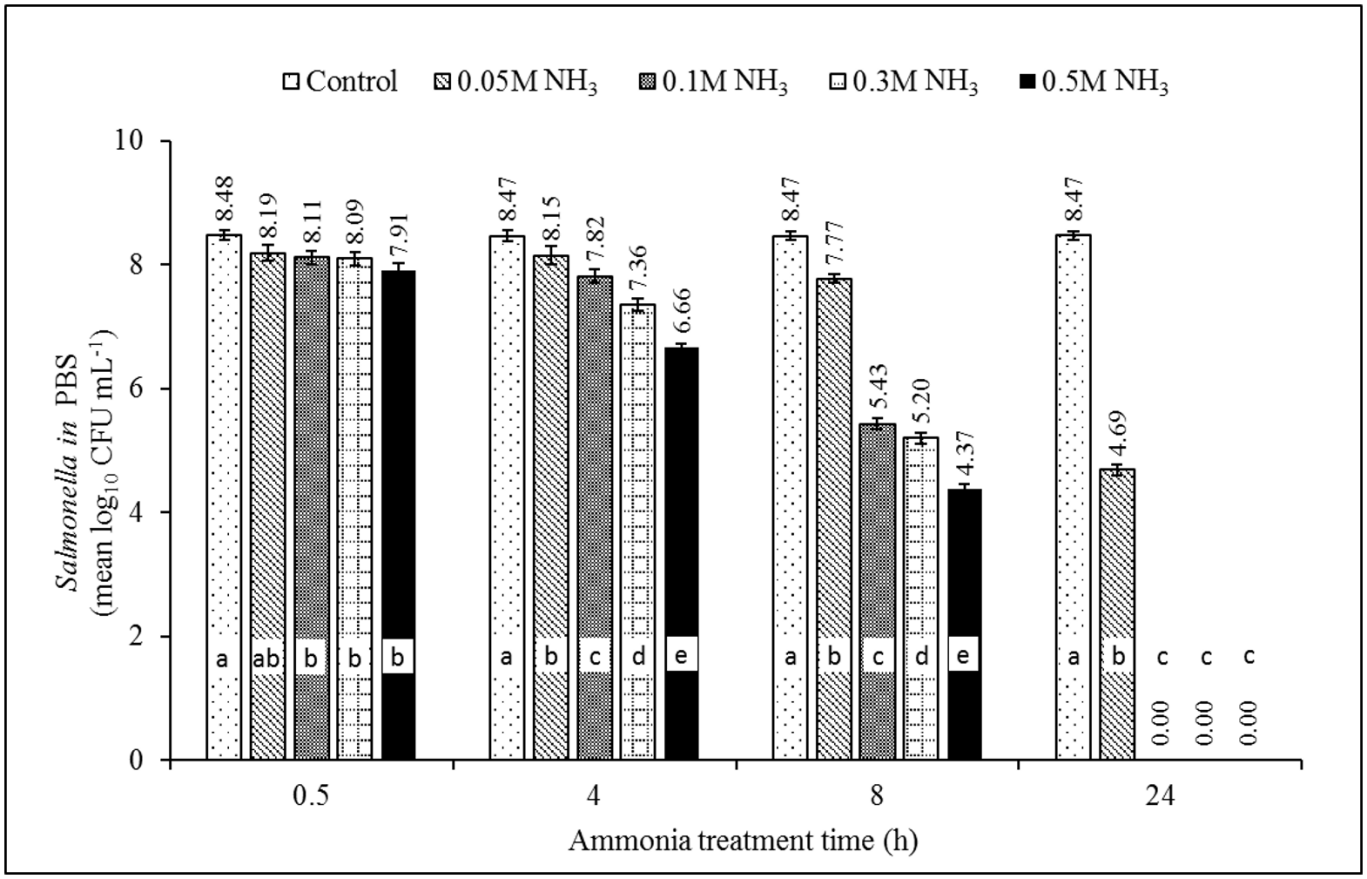
The effect of ammonia on inactivation of *Salmonella* Typhimurium χ4232 in phosphate buffer solution (PBS). Note: pH = 9.0±0.1, T = 35°C. NH_3_ concentrations of 0.052 M, 0.105 M, 0.315 M, and 0.524 M are equivalent to 734, 1,468, 4,404, and 7,340 NH_3_-N mg/L, respectively (estimated mole fraction of NH_3_-N to TAN = 0.529). Source of NH_3_ was (NH_4_)_2_SO_4_. Different letters within each treatment time indicate significant difference (*p*<0.05), n = 3.

**Fig 3:**
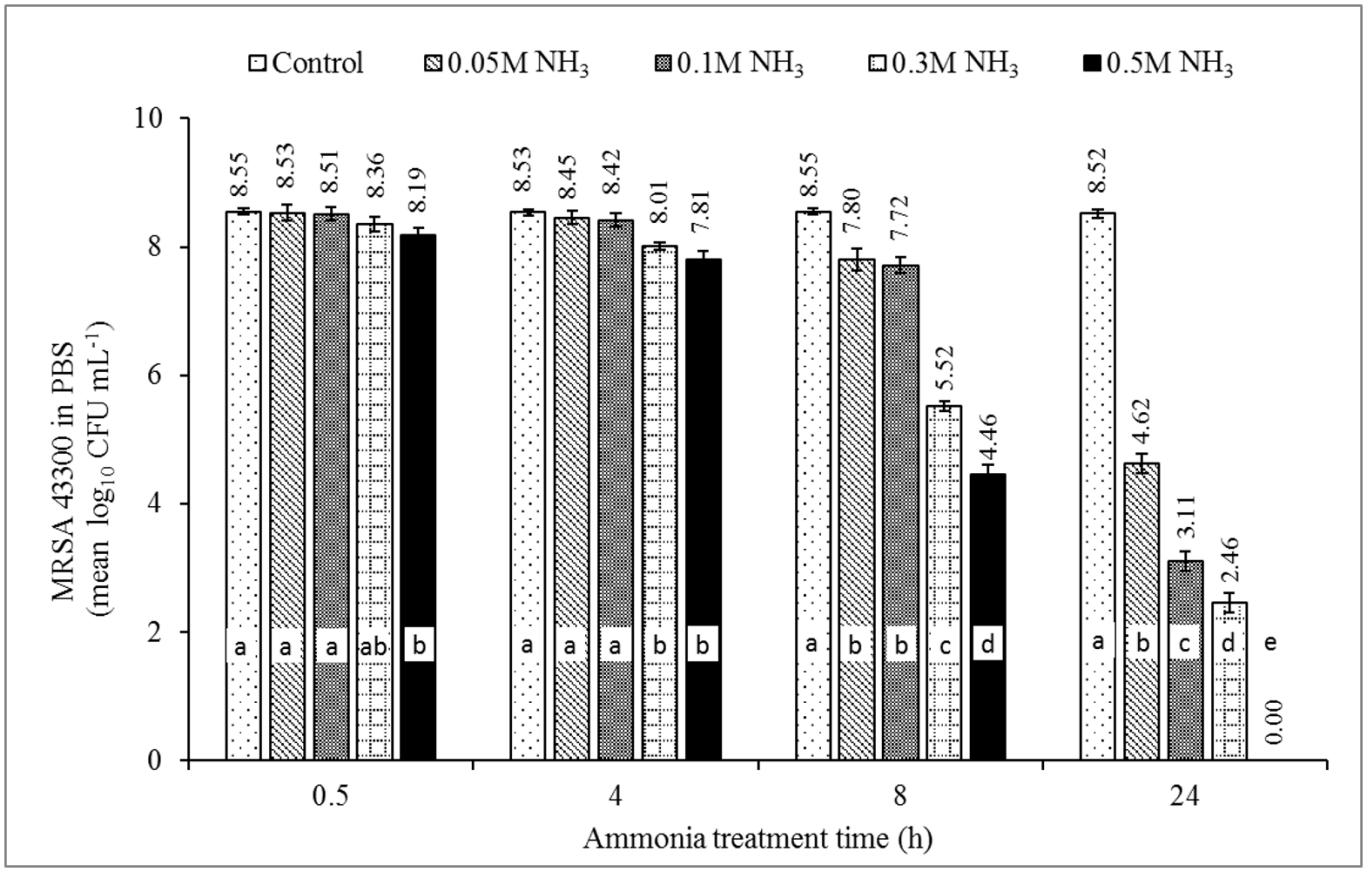
The effect of ammonia on inactivation of methicillin resistant *Staphylococcus aureus* MRSA ATCC 43300 in phosphate buffer solution (PBS). Note: pH = 9.0±0.1, T = 35°C. NH_3_ concentrations of 0.052 M, 0.105 M, 0.315 M, and 0.524 M are equivalent to 734, 1,468, 4,404, and 7,340 NH_3_-N mg/L, respectively (estimated mole fraction of NH_3_-N to TAN = 0.529). Source of NH_3_ was (NH_4_)_2_SO_4_. Different letters in each treatment time indicate significant difference (*p*<0.05), n = 3.

The effect of NH_3_ on survival of ST4232 and MRSA43300 at pH = 9.0±0.1 (NH_3_-N = 0.052 M, 0.105 M, 0.315 M, and 0.524 M) is shown in Figs 2 and 3, respectively. Growth was inhibited more effectively with greater dose and longer contact time. The statistically significant difference of the dose was observed as early as 4 h for ST4232 and at 24 h for MRSA43300. Growth ceased to occur at 24 h incubation, i.e., permitting the MIC to be estimated as defined in [56]. A visual example of plate count for *Salmonella* with treatment time is presented in S2 Fig. The MICs of ammonia were 0.105 M NH_3_-N (~1,468 mg/L of NH_3_-N), and 0.524 M NH_3_-N (~7,340 mg/L of NH_3_-N) for ST4232, and MRSA 43300, respectively (Figs 2 & 3, Table 2). The MICs are similar to those reported by Leejeerajumnean et al. [46] in culture media, i.e., 0.3 M for both ST and SA albeit for different strains, longer time (48 h), and higher incubation temperature (37°C). Lower MICs were reported in the same study for other important foodborne pathogens, i.e., 0.025 M (*E*. *feacalis*, *Listeria innocua*, *E*. *coli*) and 0.050 M (*Pseudomonas aeruginosa*) [46]. The MICs for various species of *Bacillus* ranged from 0.025 to 0.5 M [46].

**Table 2:** Minimum inhibitory concentrations (MICs) of NH_3_ (un-ionized ammonia) (24 h treatment) for pure ST4232 and MRSA43300 in PBS at pH = 9.0±0.1 and T = 35°C.

Bacteria strains	MIC [NH_3_-N] (M)	MIC [NH_3_-N] (mg/L)
***Salmonella* Typhimurium χ4232**	0.105 M	1,468
**methicillin resistant *Staphylococcus aureus* (MRSA) ATCC 43300**	0.524 M	7,340

### NH_3_ treatment after the 1^st^ barrier-treated digestate

#### Testing secondary barrier treatment in the ‘early-phase’ AeD scenario

**Fig 4:**
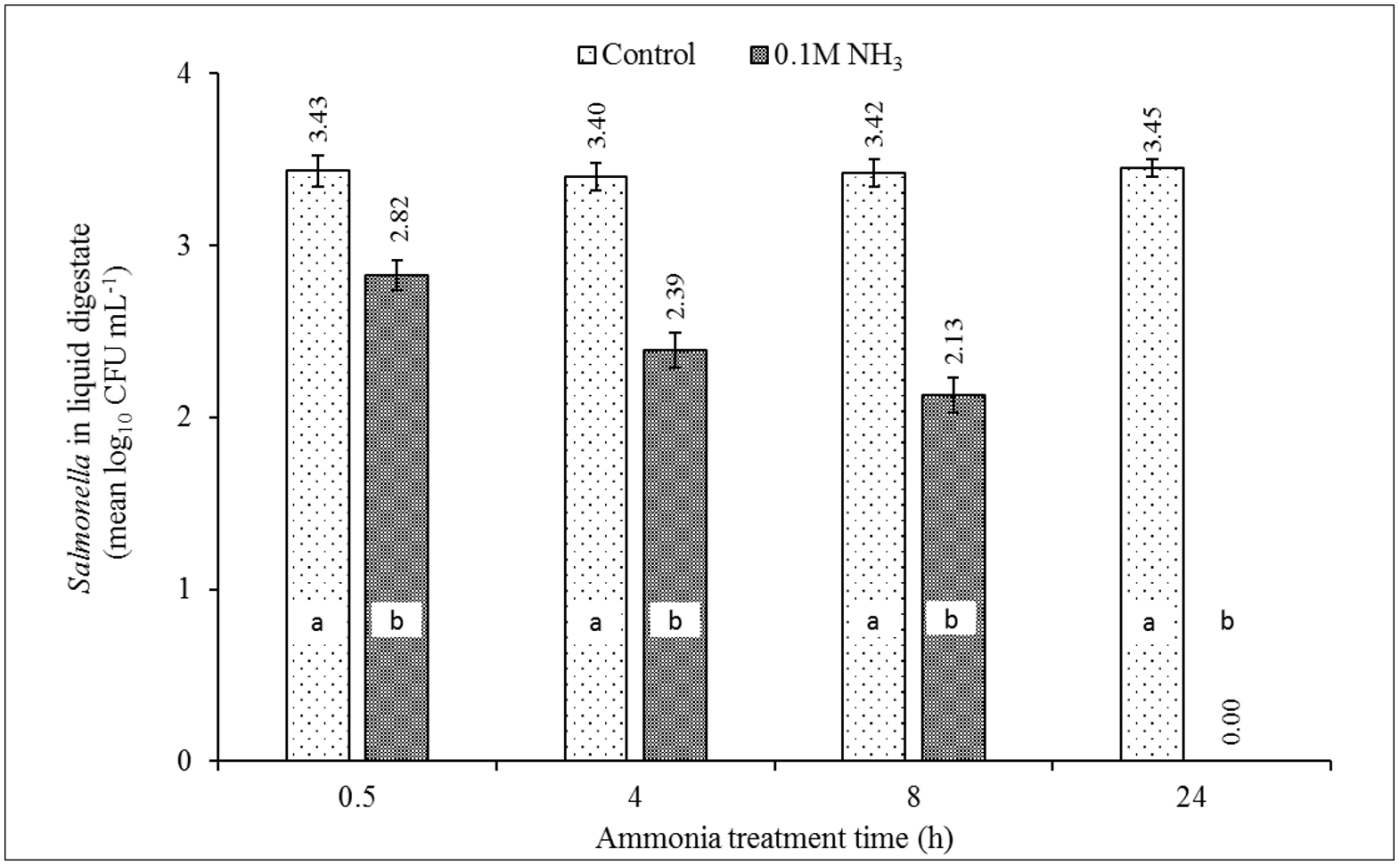
The effect of MIC of ammonia (0.105 M NH_3_; the equivalent of 1,468 NH_3_-N mg/L) on inactivation of ST4232 in digestate of week #3 of poultry carcass aerobic digestion. Source of NH_3_ was (NH_4_)_2_SO_4_. The control contained only residual TAN (0.039 M) in the digestate collected at week #3 [24]. Week #3 represents an early-phase of decomposition characterized by high BOD, highest TSS & VSS levels, and the first visual evidence of the whole carcass breakup and disintegration. Data represent the means log_10_ of measured *Salmonella* concentrations of 4 aerobic reactors ± SD. Different letters in each treatment time indicate significant difference (*p*<0.05), n = 3.

The surviving level of ST4232 at week #3 of aerobic digestion was 3.5 × 10^3^ CFU/mL while MRSA43300 was not detected [24]. The experimental results showed that the reduction in a number of surviving ST4232 from the initial (start of week 1) inoculation in digestate were 0.6, 1.0, 1.3, and 3.5 log_10_ CFU/mL after 0.5, 4, 8, and 24 h of treatment with 0.1 M NH_3_ respectively (Fig 4). The statistically significant difference for the dose was observed for all treatment times. The results were consistent with Objective (1), i.e., complete inactivation of ST4232 after 24 h of 0.105 M NH_3_ (1,468 NH_3_-N mg/L). Again, the results showed that ST4232 in the control samples were not inactivated at the high concentration of OH^-^ (pH = 9), i.e., pH did not contribute to growth inhibition (Fig 4).

#### Testing secondary barrier treatment in the ‘late-phase’ AeD scenario

**Fig 5:**
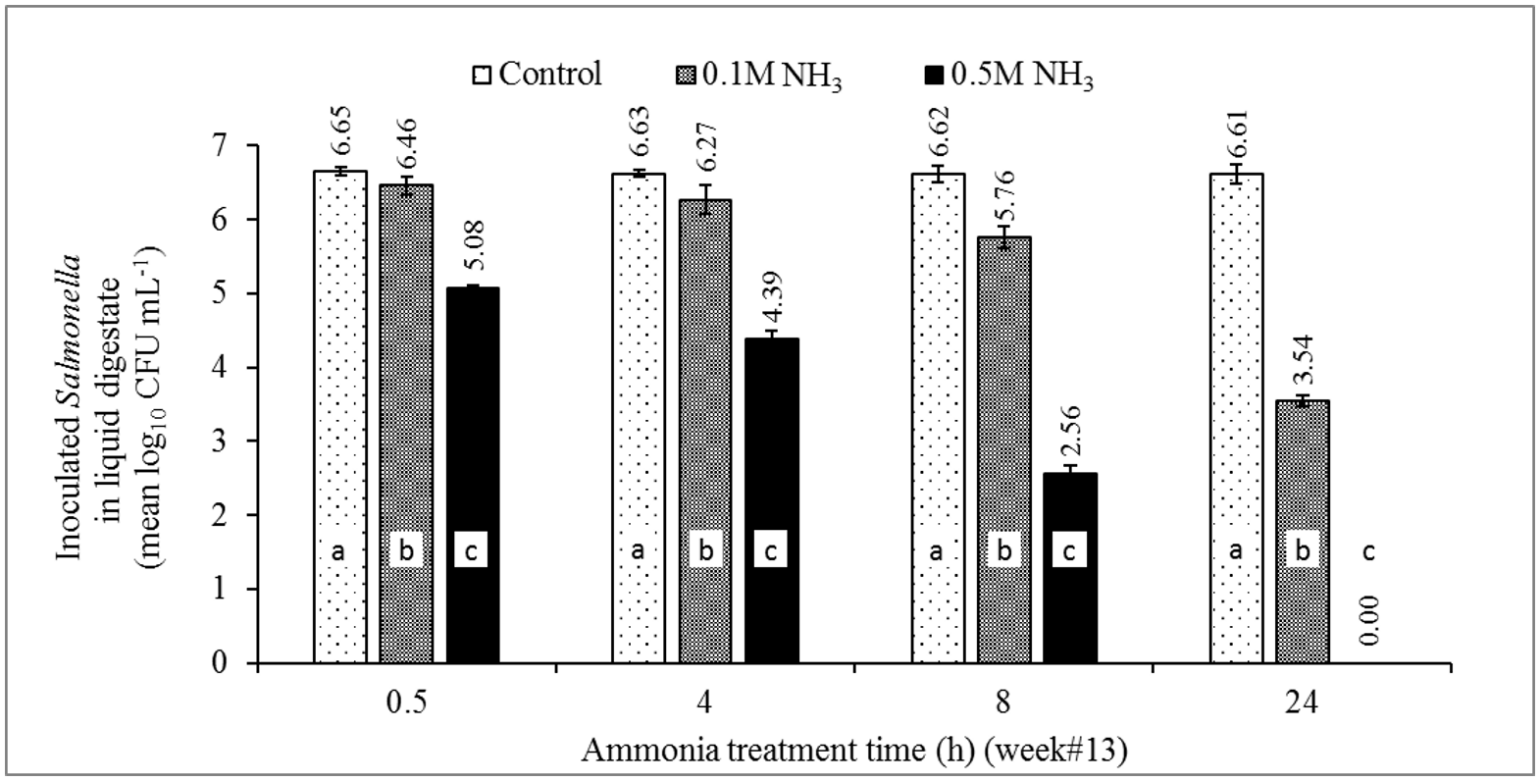
The effect of MIC of ammonia (0.105 and 0.524 M NH_3_; the equivalent of 1,468 and 7,340 NH_3_-N mg/L) on the survival of newly seeded ST4232 in digestate of week #13 poultry carcass aerobic digestion. Source of NH_3_ was (NH_4_)_2_SO_4_. The control contained only residual TAN (0.00057 M) in the digestate collected at week #13 [24]. Week #13 represents a late-phase of a complete aerobic digestion represented by lower microbial activity post the 99% reduction of BOD, TSS, and VSS. Data represents the mean log_10_ of measured *Salmonella* concentrations of 4 aerobic reactors ± SD. Different letters in each treatment time indicate significant difference (*p*<0.05), n = 3.

**Fig 6:**
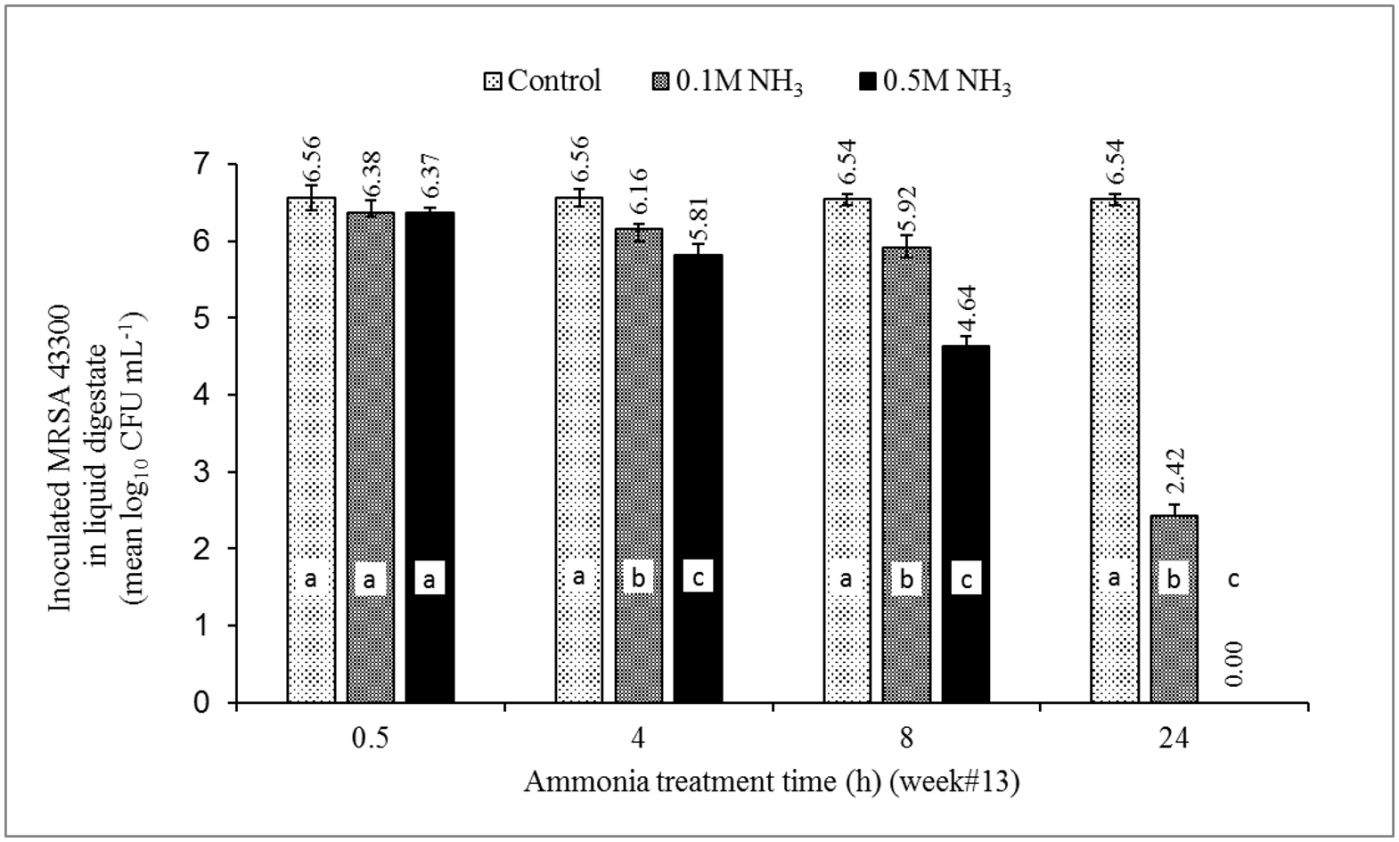
The effect of MIC of ammonia (0.105 and 0.524 M NH_3_; the equivalent of 1,468 and 7,340 NH_3_-N mg/L) on the survival of newly seeded MRSA43300 in digestate of week #13 poultry carcass aerobic decomposition. Source of NH_3_ was (NH_4_)_2_SO_4_. Week #13 represents a late-phase of a complete aerobic digestion represented by lower microbial activity post the 99% reduction of BOD, TSS, and VSS. Data represents the mean log_10_ of measured concentrations of MRSA43300 of 4 aerobic reactors ± SD. Different letters in each treatment time indicate significant difference (*p*<0.05), n = 3.

Compared to initial concentrations of inoculated bacteria, the reduction in numbers of ST4232 in digestate with NH_3_ concentrations of 0.105 M and 0.524 M ranged from 0.19–3.07, and 1.57–6.61 log_10_ CFU/mL, respectively (Fig 5). Statistically significant differences to the dose were observed for all treatment times. Likewise, the reduction in numbers of MRSA43300 ranged from 0.18–4.12, and 0.19–6.54 log_10_ CFU/mL with NH_3_ concentrations of 0.105 and 0.524 M, respectively (Fig 6). The statistically significant difference to the dose was observed for treatment times ≥ 4 h.

The required dose of ammonia to inactivate ST4232 increased from 0.105 to 0.524 M NH_3_ compared to Objective 1 (MIC) and Objective 2 (treatment of digestate, week 3 early-phase). This is also illustrated in S3 Fig comparing survival of ST4232 after NH_3_ treatment. ST4232 was most easily killed in PBS, then week #3 digestate and then week #13 digestate. Possibly either microbial population or matrix differences (early- vs. late-phase) contributed to the decreased effect of ammonia by either (1) utilization of ammonia or (2) binding of the active form of ammonia. This difference in ammonia dose for ST4232 may be a consideration when using the ammonia treatment option in a secondary barrier approach. There was very little apparent difference between surviving MSRA43300 post-NH_3_ treatment in PBS and week #13 digestate (S4 Fig).

### Mechanism of pathogen inactivation by NH_3_

It was reported that ammonia could be toxic to a variety microorganisms ranging from bacteria and viruses to mammals [57–61]. According to Sprott (1984), when NH_3_ enters the bacterial cells, it drives some protons off the cell. To maintain its internal pH, the cell takes up some protons from the outside of the cell. At the same time, it ‘sacrifices’ the potassium ion (K^+^) via efflux from the cell, and the cell dies due to the lack of this essential nutrient [62]. In this current study, elevated NH_3_ levels at pH 9.0 ± 0.1 were the prerequisite for significant reductions of the two model bacteria, ST4232 and MRSA43300. High pH = 9 and the resulting high molecular fractions of NH_3_ to TAN used in this study are believed to provide efficient means of bacterial inactivation. The TAN, by itself, does not appear to be the reason for pathogen inactivation. A sufficient source of NH_3_ (e.g., (NH_4_)_2_SO_4_) must be accompanied by elevated pH (e.g., by the addition of KOH) to sustain an NH_3_ fraction of TAN as shown in S1 Table. Increased temperature can also boost the NH_3_ fraction (S1 Table). S1 Table provides practical information on how to achieve NH_3_ concentrations required to meet MICs for model strains of ST4232 and MRSA4330 under varied conditions of pH (4 to 12) and temperature (mesophilic temperature = 35°C, room temperature = 20°C).

### Potential use of NH_3_ treatment for other pathogens of interest

The effect of NH_3_ as a secondary barrier treatment for inactivation of two model bacteria representing gram-positive (MSRA43300) and gram-negative (ST4232) pathogens was studied. The results (Objective 1) were in agreement with earlier observations that the gram-negative bacteria are more susceptible to ammonia at higher pH than gram-positive bacteria [52]. The results from Objective 2 are of particular utility for further development of the burial-AeD hybrid concept and the use of NH_3_ as a secondary barrier treatment. Gwyther et al. [26] reported that bacterial counts of *S*. *enterica*, *E*. *faecalis*, *C*. *jejuni*, *C*. *coli*, and *E*. *coli* O157 in sheep carcass components were decreased significantly (>5-log 10). However, *E*. *faecalis* (gram-positive) remained detectable until the end of 3 months of the trial [26]. The results of this study suggest that higher NH_3_ dose is generally needed to inactivate gram-positive bacteria. This observation is in general agreement with reports on ammonia effects on bacterial population in manures, feed and meat [48–53].

The results can be also informative to consider the potential usefulness of NH_3_ in the context of improving biosecurity in livestock and poultry production systems. Himathongkham et al. [48] reported similar survival rates for *S*. *typhimurium* and *E*.*coli* (0157:H7) (both gram-negative bacteria) in stored cow manure and slurry with controlled and elevated pH. Himathongkham et al. [49] reported similar significant reduction for *S*. *typhimurium*, *E*.*coli* (0157:H7), and ~half as effective reduction of *L*. *monocytogenes* (a gram-positive bacteria) in chicken manure gassed with NH_3_. Niebuhr and Dickson reported on the impact of pH enhancement with ammonia gas to pH ~ 9.6 on populations of *Salmonella*, *L*. *monocytogenes*, and *E*. *coli* O157:H7 in boneless lean beef trimmings [52]. Ammoniation reduced *E*.*coli* (0157:H7) by ~3-log10 CFU/g and Salmonellae by ~4.5-log10 CFU/g, and only ~0.5-log10 CFU/g of *L*. *monocytogenes* [52]. Effectiveness of NH_3_ treatment of inoculated animal feed was also studied by Tajkarimi et al. [53]. *C*. *jejuni*, *E*.*coli* (0157:H7), *Y*. *enterocolitica* and *L*. *monocytogenes* were consistently reduced at or above 5-log10 [53].

## Conclusions

The MICs (Objective 1) for ST4232 and MRSA43300 were 0.1 M NH_3_ (~1,468 mg/L of NH_3_-N) and 0.5 M NH_3_ (~7,340 mg/L of NH_3_-N) concentrations, respectively. Inactivation was increased by increasing NH_3_ concentration and/or treatment time. Although the chemistry and microbiology of digestate are complex, the effectiveness of NH_3_ treatment of digestate (Objective 2) was consistent with the MICs determined in sterile saline solution except ST4232 in the late-phase AeD scenario where the MIC was 5x greater. Both pathogens, however, were completely inactivated after 24 h. A sufficient source of NH_3_ (e.g., (NH_4_)_2_SO_4_) must be accompanied by elevated pH (e.g., by the addition of KOH) to sustain an NH_3_ fraction of TAN. Increased temperature can also boost the NH_3_ fraction. High pH (≥9) and the resulting high molecular fraction of NH_3_ are believed to provide efficient means of bacterial inactivation. Further work is warranted to determine what other important pathogen species important in livestock production systems could be practically inactivated with NH_3_. Results of this proof-of-concept study show that the secondary barrier approach can reduce the risks of pathogen contamination of shallow groundwater pollution when the temporary liner employed in the burial-AeD hybrid carcass disposal concept ultimately ruptures or if digestate is pumped out of the lined trench. If found to be true in the field, post-digestion treatment of digestate with NH_3_ could conceivably become a useful addition to the in-trench burial-AeD disposal method, thereby making it more biosecure.

## Supporting information

S1 Fig The effect of ammonia (NH_3_ source was NH_4_Cl) on inactivation of *Salmonella* Typhimurium χ4232 in sterile saline solution. Note: pH = 5.8 (Control, T = 21.3°C), 4.6 (3.7 M TAN, T = 15.9°C), 4.5 (5.6 M TAN, T = 13.6°C), and 4.4 (7.5 M TAN, T = 12.5°C). NH_3_-N fractions of TAN were extremely low and ranged from 0 to 0.001. Different letters in each treatment time indicate significant difference (p<0.05), n = 3.
(TIF)

S2 Fig Visual total counts of Salmonella in control plates versus ammonia treatments with 3 different treatment times (4, 8, and 24 h). (TIF)

S3 Fig Comparison of ST4232 survival after NH_3_ treatment of digestate at late-phase, early-phase, and treatment of pure culture. (TIF)

S4 Fig Comparison of MSRA43300 survival after NH_3_ treatment of digestate at late-phase, and treatment of pure culture. (TIF)

S1 Table The TAN concentrations required to meet minimum inhibitory concentrations (MICs) of NH_3_ for model strains of ST4232 and MRSA43300 under varied conditions of pH (4 ~ 12) and temperature (mesophilic temperature = 35°C, room temperature = 20°C). (PDF)
