# Supplementary figures and images for: Efficacy of NH3 as a secondary barrier treatment for inactivation of Salmonella Typhimurium and methicillin-resistant Staphylococcus aureus in digestate of animal carcasses: Proof-of-concept
Source: PLoS One. 2017 May 5;12(5):e0176825. doi: 10.1371/journal.pone.0176825 (PMC5419515; doi:10.1371/journal.pone.0176825)

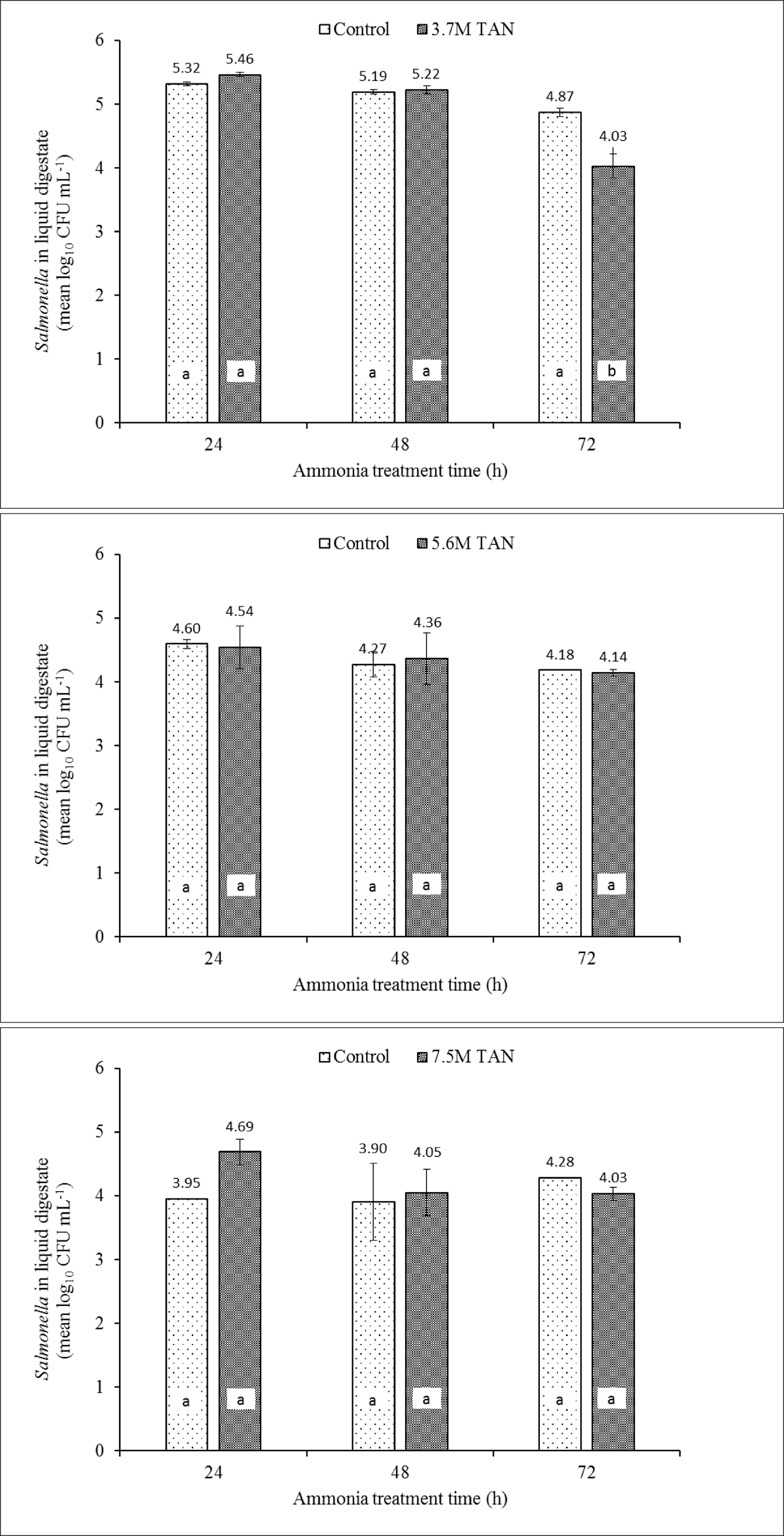

Supplement: S1 Fig — Note: pH = 5.8 (Control, T = 21.3°C), 4.6 (3.7 M TAN, T = 15.9°C), 4.5 (5.6 M TAN, T = 13.6°C), and 4.4 (7.5 M TAN, T = 12.5°C). NH3-N fractions of TAN were extremely low and ranged from 0 to 0.001. Different letters in each treatment time indicate significant difference (p<0.05), n = 3. (TIF) [file pone.0176825.s001.tif]

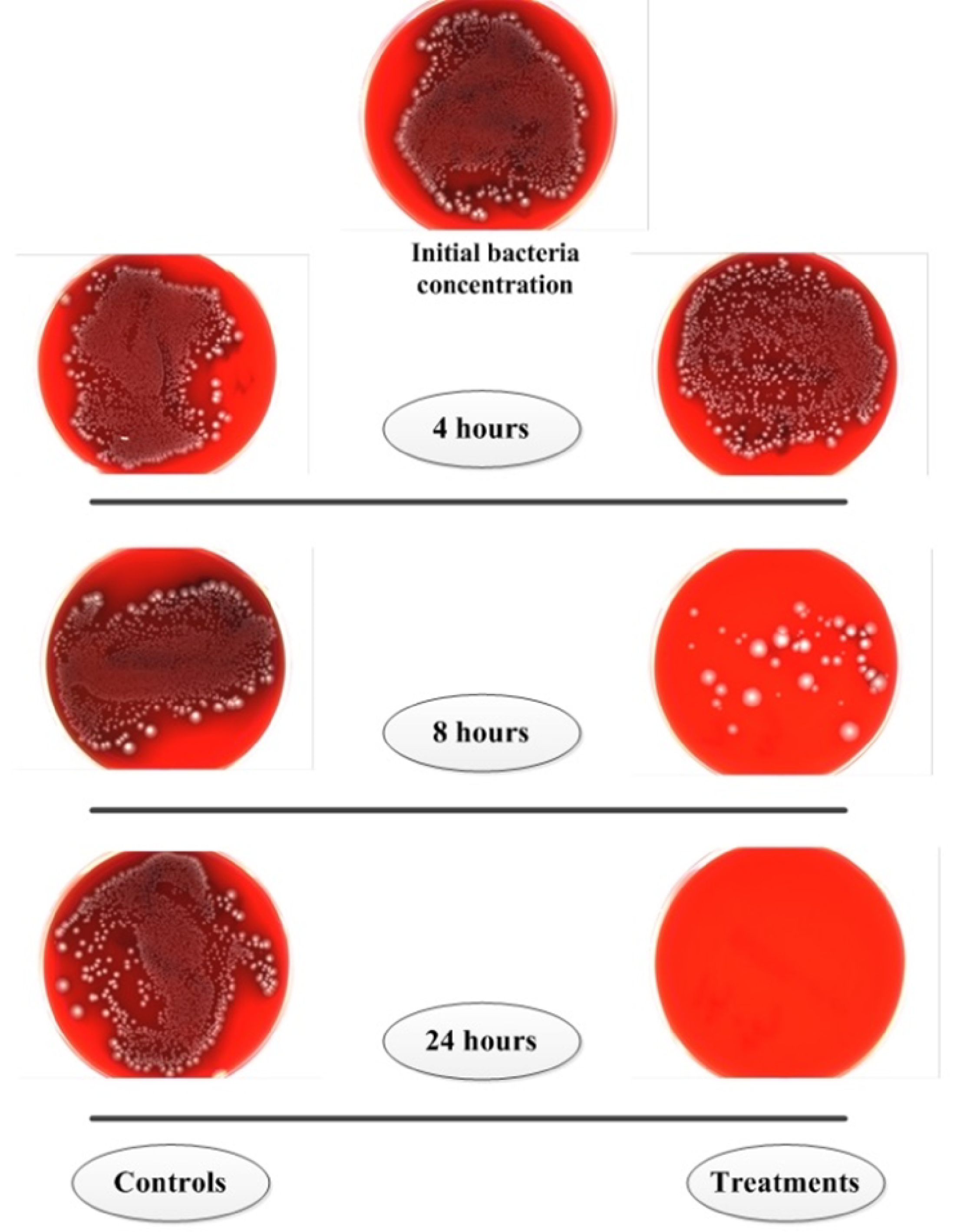

Supplement: S2 Fig — (TIF) [file pone.0176825.s002.tif]

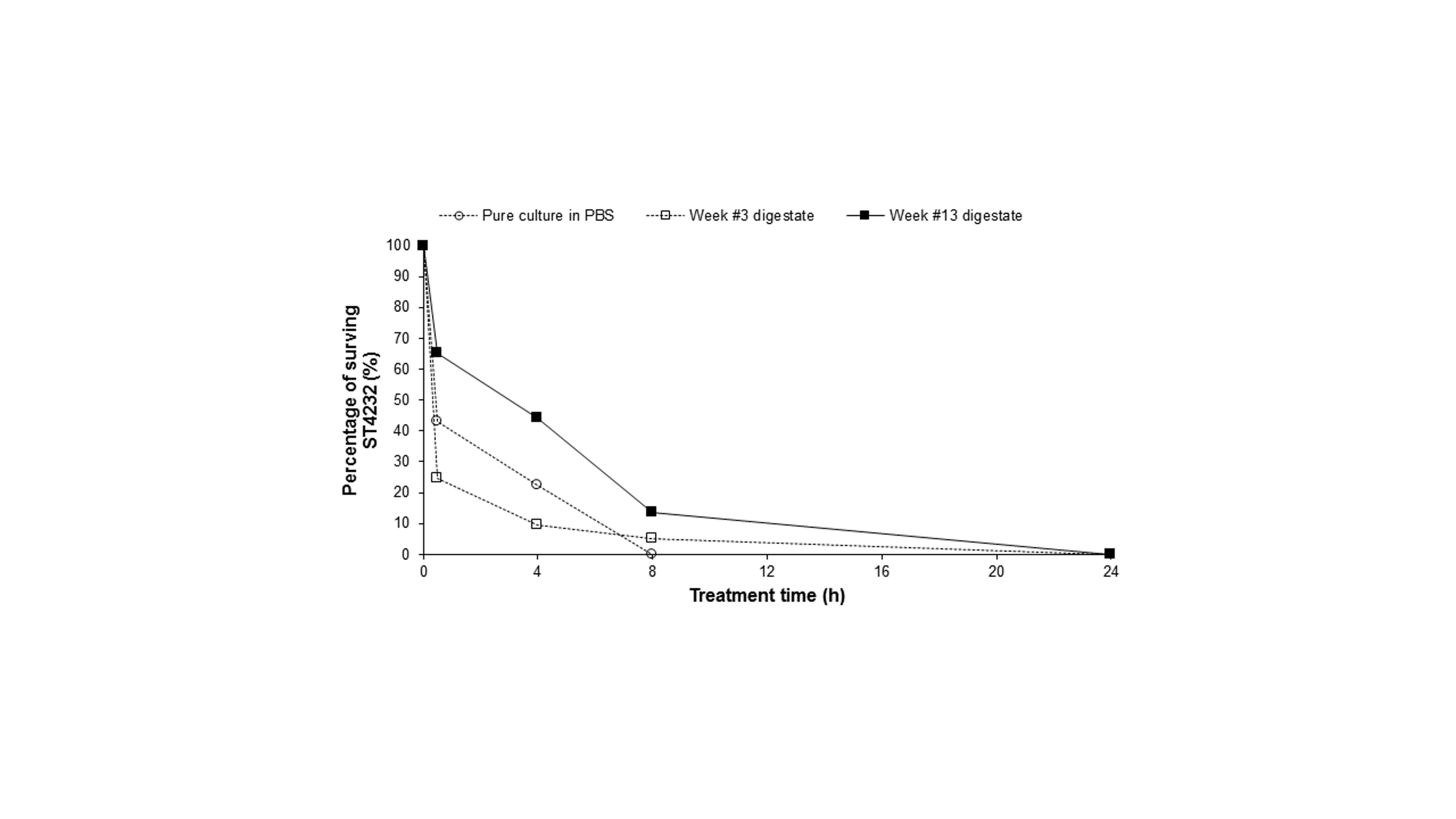

Supplement: S3 Fig — (TIF) [file pone.0176825.s003.tif]

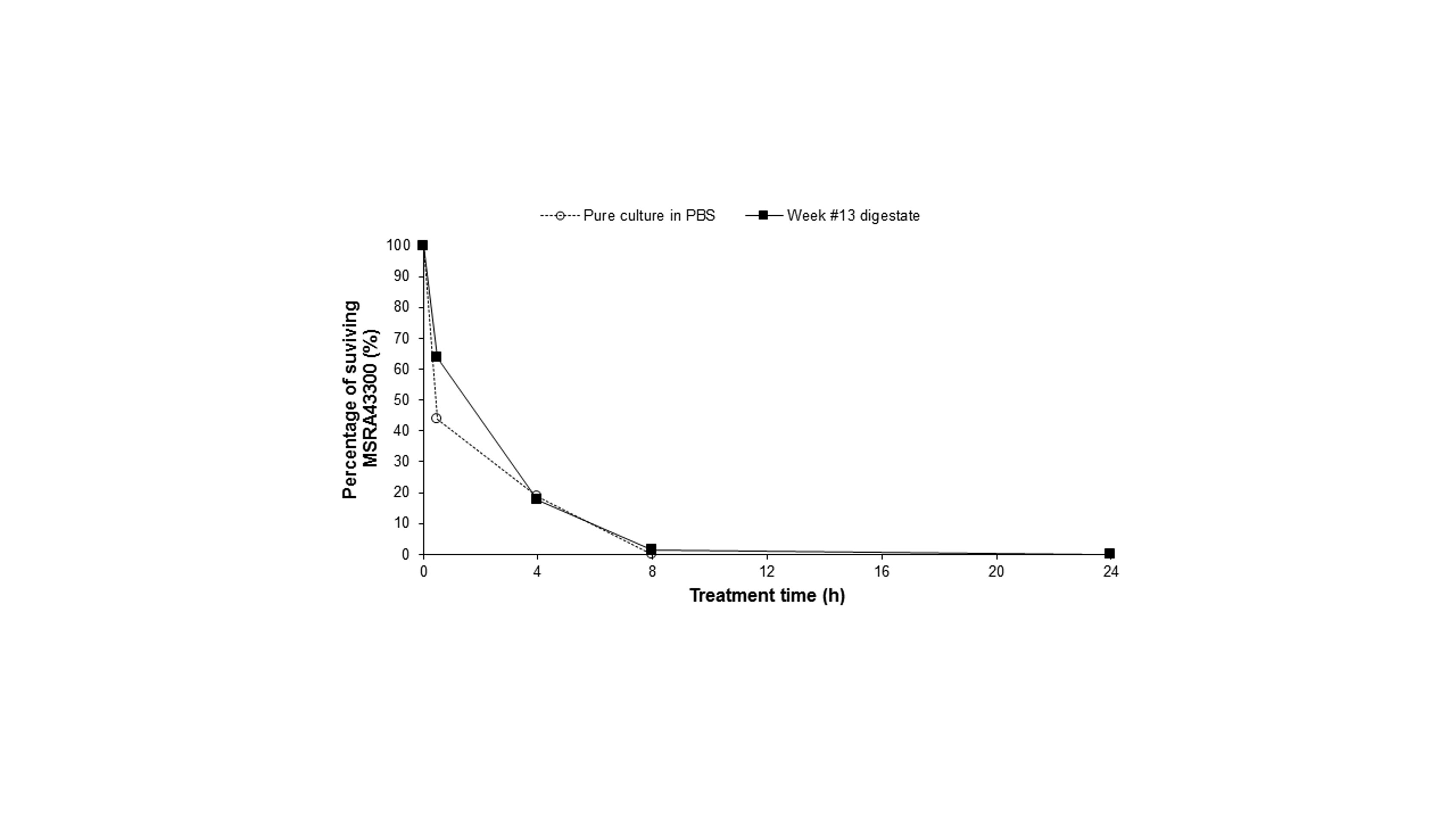

Supplement: S4 Fig — (TIF) [file pone.0176825.s004.tif]
