# Supplementary material for: Efficacy of NH3 as a secondary barrier treatment for inactivation of Salmonella Typhimurium and methicillin-resistant Staphylococcus aureus in digestate of animal carcasses: Proof-of-concept
Source: PLoS One. 2017 May 5;12(5):e0176825. doi: 10.1371/journal.pone.0176825 (PMC5419515; doi:10.1371/journal.pone.0176825)
Supplement: S1 Table — (PDF) [file pone.0176825.s005.pdf]

S1 Table. The TAN concentrations required to meet minimum inhibitory concentrations (MICs) of NH_3 for model strains of ST4232 and MRSA43300 under varied conditions of pH (4 ~ 12) and temperature (mesophilic temperature = 35 °C, room temperature = 20 °C).

**pH**			**4**	**5**	**6**	**7**	**8**	**9**	**10**	**11**	**12**
**35 °C**	**% of NH_3-N in TAN**		0.001	0.011	0.113	1.1	10.1	**53.0**	91.9	99.1	99.9
	**MIC for ST4232** (1,468 NH_3-N mg/L)	**TAN** (g/L)	146,794	13,345	1,299	132	15	2.8	1.6	1.48	1.47
		**M TAN**	10,485	953	92.8	9.4	1.0	**0.20**	0.11	0.11	0.10
	**MIC for MRSA43300** (7,340 NH_3-N mg/L)	**TAN** (g/L)	146,794	66,725	6,495	659	72	13.8	7.9	7.41	7.35
		**M TAN**	10,485	4,766	464	47	5.2	**1.0**	0.57	0.53	0.52
**20 °C**	**% of NH_3-N in TAN**		0	0.004	0.04	0.4	3.8	**28.4**	79.9	97.5	99.7
	**MIC for ST4232** (1,468 NH_3-N mg/L)	**TAN** (g/L)	-	36,698	3,669	371	38	5.2	1.8	1.51	1.47
		**M TAN**	-	2,621	262.1	26.5	2.7	**0.37**	0.13	0.11	0.11
	**MIC for MRSA43300** (7,340 NH_3-N mg/L)	**TAN** (g/L)	-	183,493	18,349	1,858	192	25.8	9.2	7.53	7.36
		**M TAN**	-	13,107	1,311	133	13.7	**1.8**	0.66	0.54	0.53
